# Anti-TIM3 chimeric antigen receptor-natural killer cells preferentially target primitive acute myeloid leukemia cells with minimal fratricide and exhaustion

**DOI:** 10.1186/s40164-024-00534-2

**Published:** 2024-07-11

**Authors:** Phatchanat Klaihmon, Parinya Samart, Yon Rojanasakul, Surapol Issaragrisil, Sudjit Luanpitpong

**Affiliations:** 1 Siriraj Center of Excellence for Stem Cell Research, Faculty of Medicine Siriraj Hospital, Mahidol University, 2 Siriraj Hospital, Bangkoknoi, Bangkok, 10700 Thailand; 2 Department of Pharmaceutical Sciences, West Virginia University, Morgantown, WV USA; 3 Present Address: Division of Hematology, Department of Medicine, Faculty of Medicine Siriraj Hospital, Mahidol University, Bangkok, Thailand; 4 Blood Products and Cellular Immunotherapy Research Group, Faculty of Medicine Siriraj Hospital, Mahidol University, Bangkok, Thailand

**Keywords:** Acute myeloid leukemia, Immunotherapy, Chimeric antigen receptor, TIM3, Natural killer cell, Exhaustion

## Abstract

Acute myeloid leukemia (AML) is an aggressive and genetically heterogeneous disease with poor clinical outcomes. Refractory AML is common, and relapse remains a major challenge, attributable to the presence of therapy-resistant leukemic stem cells (LSCs), which possess self-renewal and repopulating capability. Targeting LSCs is currently the most promising avenue for long-term management of AML. Likewise, chimeric antigen receptor (CAR)-natural killer (NK) cells have emerged as a promising alternative to CAR-T cells due to their intrinsic potential as off-the-shelf products and safer clinical profiles. Here, we introduced a third-generation CAR harboring TIM3 scFv, CD28, 4-1BB, and CD3ζ (CAR-TIM3) into human NK-92 cells, the only FDA-approved NK cell line for clinical trials. TIM3 was chosen as a target antigen owing to its differential expression in LSCs and normal hematopoietic stem/progenitor cells (HSPCs). The established CAR-TIM3 NK-92 cells effectively targeted TIM3 and displayed potent anti-tumor activity against various primitive AML cells, subsequently causing a reduction in leukemic clonogenic growth in vitro, while having minimal effects on HSPCs. CAR-TIM3 NK-92 cells significantly reduced leukemic burden in vivo and interestingly suppressed the engraftment of AML cells into the mouse liver and bone marrow. Surprisingly, we found that CAR-TIM3 NK-92 cells expressed relatively low surface TIM3, leading to a low fratricidal effect. As TIM3 and PD-1 are immune checkpoints involved in NK cell dysfunction, we further tested and found that CAR-TIM3 NK-92 cells are beneficial for alleviating NK cell exhaustion. Our findings highlight the potential application of CAR-TIM3 NK cells for cellular immunotherapy for TIM3^+^ AML.

**Supplementary Information:**

The online version contains supplementary material available at 10.1186/s40164-024-00534-2.

**To the editor,**

Current treatment for acute myeloid leukemia (AML) patients relies mainly on standard 7 + 3 chemotherapy followed by allogeneic hematopoietic stem cell transplantation, which is not applicable for unfit/elderly patients [1]. Although complete remission may be achieved, the majority of patients will eventually experience a relapse, which is likely due to the presence of leukemic stem cells (LSCs) [2]. Analogous to hematopoietic stem cells (HSCs), LSCs sit at the apex of the hierarchy of malignant hematopoiesis to self-renew and generate more mature progenies. Targeting LSCs is currently the most promising avenue for the long-term control of the disease, but a challenge arises from the fact that LSCs share a mutual immunophenotype with normal HSCs. TIM3 (encoded by *HAVCR2*) appears to be a promising clinical target that is associated with unfavorable prognosis in AML patients [3, 4]. Our database analyses validated the differentially expression of *HAVCR2* between HSCs and AML blasts regardless of genetic and phenotypic characteristics and between LSCs and HSCs (Additional file 1: Methods; Additional file 2: Figs. S1 and S2). Flow cytometric analysis was also performed to confirm that surface TIM3 is highly expressed in LSCs and LPCs, while minimally expressed in neutrophils, monocytes, NK, and T cells obtained from either AML or normal whole blood sample (Additional file 2: Fig. S3).

In recent years, chimeric antigen receptor (CAR)-T cell therapy has shown great promise for treating patients with hematological malignancies, especially those with relapsed/refractory CD19^+^ neoplasms [5]. In 2021, Lee et al. discovered that anti-TIM3 CAR-T cells had potent anti-AML activity in the mouse models [6]. However, major limitations of CAR-T cell therapy include life-threatening complications such as graft versus host disease (GvHD), cytokine-releasing syndrome (CRS), and immune effector cell-associated neurotoxicity syndrome (ICANS) [7]. CAR-NK cells represent a more feasible cellular immunotherapy due to their potent anti-tumor activity without prior sensitization and safer clinical profiles [8]. Herein, we engineered human NK-92 cells, the only FDA-approved NK cell line for clinical trials [8], with a third-generation CAR harboring a signal peptide, anti-TIM3 scFv (clone TSR-022), CD8 hinge, CD28 transmembrane, and intracellular costimulatory domains of CD28 and 4-1BB joined to CD3ζ signaling (CAR-TIM3). After rounds of enrichment, CAR-TIM3 NK-92 cells were validated for CAR expression by RT-qPCR and Western blotting (Fig. 1A) and for target antigen-based binding and activation (Fig. S4). To our surprise, we found a remarkable decrease in surface TIM3 in CAR-TIM3 NK-92 cells when compared to wild type (WT) NK-92 cells (Fig. 1B), which was likely attributed to its low self-killing event, unlike WT NK-92 cells that underwent apoptosis following CAR-TIM3 NK cell exposure (Fig. 1C). CAR-TIM3 NK-92 cells preferentially exert potent anti-leukemic function, e.g., at a relatively low E:T ratio of 1:10 at 4 h, in CD34^+^ primitive AML cells, but minimally harm normal hematopoietic stem/progenitor cells (HSPCs) (Fig. 1D and E). We also showed that CAR-TIM3 NK-92 cells significantly inhibited leukemic colony-forming cells, the functional progenitors that support the self-renewal of AML blasts (Fig. 1F), suggesting that CAR-TIM3 NK cells may result in the long-term control of primitive AML cells. We further validated the selective cytotoxicity of CAR-TIM3 NK-92 cells against TIM3^+^ AML cells using primary AML cells (Fig. S5) and TIM3-overexpressed (O/E) U937 (FAB M5) and HEL92.1.7 (M6) cells that naturally lack TIM3 (Fig. 1G; Fig. S6). We unexpectedly found that CAR-TIM3 NK-92 cells exhibited greater anti-tumor activity against mock (TIM3^−^) AML cells than WT NK-92 cells with no known mechanisms, though we observed that the former had higher basal IFN-γ level and NK activating receptors (Figs. S7 and S8). Importantly, our CAR-TIM3 construct was proven to be effective in transducing peripheral blood NK cells (Fig. S9).

**Fig. 1:**
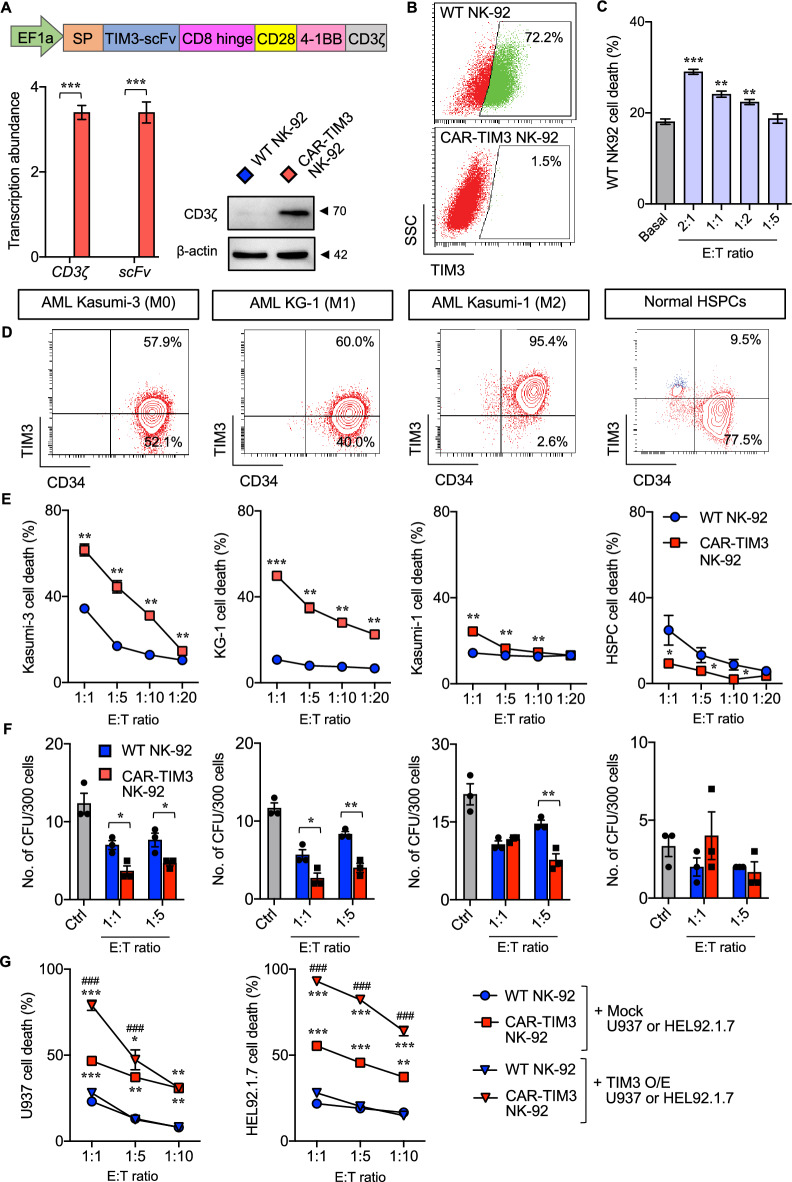
CAR-TIM3 NK-92 cells effectively target TIM3^+^ primitive AML cells. **A** (upper) Schematic illustration of the third generation anti-TIM3 CAR construct (CAR-TIM3). (lower) (left) RT-qPCR analysis of CD3ζ and anti-TIM3 scFv fragment and (right) Western blotting of CD3ζ protein in WT and CAR-TIM3 NK-92 cells. **B** Flow cytometric analysis showing reduced surface TIM3 in CAR-TIM3 NK-92 cells comparing to WT cells. **C** NK cell cytotoxicity was performed by labeling target (T) cells with PKH67 dye before exposure to unlabeled effector (E) cells. Percentages of total cell death of PKH67-labeled WT NK-92 cells, comprising annexin V- and/or 7-AAD-positive cells, after exposure to unlabeled CAR-TIM3 NK-92 cells at different E:T ratios for 4 h by annexin V/7-AAD assay. **p < 0.01; ***p < 0.001 vs basal apoptosis in WT NK-92 cells; one-way ANOVA. **D** Flow cytometric analysis of CD34 and TIM3 expression in primitive AML cells, including Kasumi-3, KG-1, and Kasumi-1 cells, and normal HSPCs. **E** Percentages of total cell death of PKH67-labeled AML cells or HSPCs after exposure to either unlabeled WT or CAR-TIM3 NK-92 cells for 4 h by annexin-V/7-AAD assay. Basal death rate (without NK cells) was subtracted from all data shown. **p < 0.01, ***p < 0.001 vs WT NK cells at the same E:T ratio; Mann–Whitney U-test. **F** Numbers of colony formation unit (CFU) of AML cells or HSPCs after incubation with either WT or CAR-TIM3 NK-92 cells under colony formation assay for 10 days. *p < 0.05, **p < 0.01, ***p < 0.001 vs WT NK cells at the same E:T ratio; Mann–Whitney U-test. **G** CAR-TIM3 NK-92 cells preferentially target TIM3^+^ AML cells. Percentages of total cell death of PKH67-labeled WT (mock) or TIM3 overexpressed (O/E) AML cells after exposure to either WT or CAR-TIM3 NK-92 cells for 4 h by annexin-V/7-AAD assay are shown. *p < 0.05, **p < 0.01, ***p < 0.001 vs WT NK-92 cells with mock AML cells; ^###^*p* < 0.001 vs CAR-TIM3 NK-92 cells with mock AML cells; Mann–Whitney U-test

NK cells are known for their short in vivo lifespan of approximately 2 weeks [9]. Multiple infusions of CAR NK-92 cells targeting CD33 at doses up to 5 billion cells per patient were shown to be safe in AML patients [10]. Repeated injection of CAR-TIM3 NK-92 cells efficiently reduced tumor cell burden (Fig. 2A–D), and liver and bone marrow engraftment in the xenograft mouse model of AML (Fig. 2E and F). As bone marrow is known as a primary compartment where AML blasts and LSCs accumulate, our findings strongly indicated that CAR-TIM3 NK-92 cells effectively suppressed leukemic growth in vivo. TIM3 is also used as an important marker for exhausted T cells and NK cell dysfunction [11, 12]. We further disclosed the beneficial effects of CAR-TIM3 NK-92 cells, compared to those of WT NK cells, on NK function and exhaustion based on the following observations: (i) CAR-TIM3 transgene downregulated surface TIM3, which mediates NK cytotoxicity against AML cells; (ii) the CAR-TIM3 directly prevented NK exhaustion phenotype, as evaluated by TIM3^+^PD-1^+^ coexpression, upon AML exposure (Fig. 2G; (see also Fig. S10 for TIM3 knockdown experiments). Together, our findings highlight the potential application of CAR-TIM3 NK cells with preserved NK active function that preferentially target LSCs and may lead to the long-term control of AML.

**Fig. 2:**
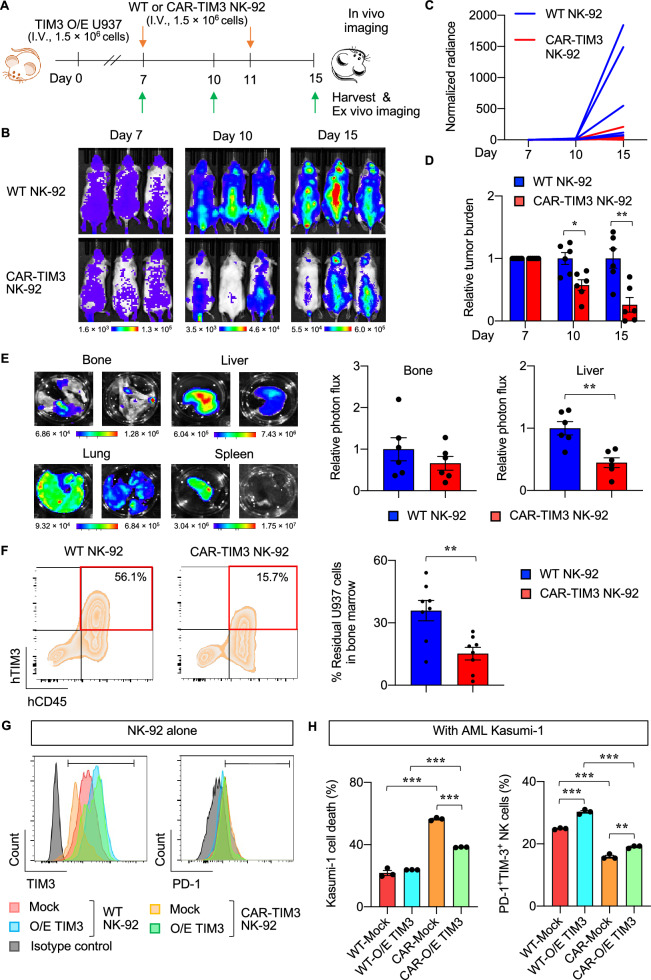
CAR-TIM3 NK-92 cells effectively reduce AML tumor burden and biodistribution in vivo. **A** Timeline for inoculation of 1.5 × 10^6^ Luc-labeld TIM3-overexpressed U937 cells, two doses of 1.5 × 10^6^ NK cells, and tumor imaging in NOD SCID gamma (NSG) mice. **B** Representative in vivo bioluminescence imaging of mice taken at days 7, 10, and 15 after tumor inoculation. **C** Quantification of whole-body signals in different mice (n = 6 per group) after receiving WT or CAR-TIM3 NK-92 cells normalized to their initial signals before NK cell treatment at day 7. **D** Relative AML tumor burden was calculated by normalization of signals (C) in CAR-TIM3 NK-92 group to those of WT NK-92 group obtained on the same day. *p < 0.05, **p < 0.01 vs WT NK-92 group; Mann–Whitney U-test. **E** (left) Representative ex vivo bioluminescence imaging of isolated organs obtained from NSG mice at the end of experiment. (right) Quantification of signals indicated a significant reduction of tumor burden in liver of mice receiving CAR-TIM3 NK-92 cells. **p < 0.01 vs WT NK-92 group; Mann–Whitney U-test. **F** Flow cytometric analysis of AML cell engraftment in isolated bone marrow based on the detection of human TIM3^+^CD45^+^ cells. Representative plots (left) and quantification (right) are shown. **p < 0.01 vs WT NK-92 group; Mann–Whitney U-test. **G**, **H** CAR-TIM3 NK cells improved NK cytotoxicity in part by reducing NK cell exhaustion. **G** Flow cytometric analysis of surface TIM3 and PD-1 in WT and CAR-TIM3 NK-92 cells transduced with TIM3 transgene (O/E TIM3) or vector control (mock) to first identify the functional role of TIM3 in NK exhaustion. **H** (left) Percentages of total cell death of PKH67-labeled Kasumi-1 cells as evaluated by annexin-V/7-AAD assay after exposure to indicated NK cells at E:T ratio of 1:1 for 4 h. (right) Percentages of TIM3 and PD-1 coexpression in indicated NK-92 cells upon exposure to Kasumi-1 cells. ***p < 0.001 vs indicated NK cells; Mann–Whitney U-test

### Supplementary Information

**Additional file 1:** Methods.

**Additional file 2: Fig. S1** Analysis of *HAVCR2* gene expression in clinical AML specimens using available public databases. **Fig. S2** Ranked gene list correlation profile for LSCs versus LPCs or normal HSCs by Gene Set Enrichment Analysis (GSEA) using GSE24006 dataset. **Fig. S3** Flow cytometric analysis of surface TIM3 expression in different subpopulations of leukocytes obtained from AML and normal whole blood samples. **Fig. S4** Validation of CAR expression in NK-92 cells based on target antigen-based binding and activation. **Fig. S5** Anti-AML activity of CAR-TIM3 NK92 cells against primary AML cells. **Fig. S6** Overexpression of TIM3 in AML cell lines with relatively more mature phenotype. **Fig. S7** Pro-inflammatory cytokines released by CAR-TIM3 NK-92 cells upon AML exposure. **Fig. S8** Flow cytometric analysis of surface NK cell activating receptors, including Nkp44, Nkp46, and NKG2D, in WT and CAR-TIM3 NK-92 cells. **Fig. S9** Anti-AML activity of peripheral blood-derived CAR-TIM3 NK cells against various AML cells. **Fig. S10** TIM3 mediates NK cytotoxicity against primary AML cells.

## Data Availability

The datasets generated and/or analyzed during the current study are available from the corresponding author on reasonable request. Additional file information is available in Additional files 1 and 2.

## Abbreviations

AML: Acute myeloid leukemia
CAR: Chimeric antigen receptor
CAR-TIM3: Third-generation CAR targeting TIM3
CFU: Colony-forming unit
CRS: Cytokine-releasing syndrome
E:T: Effector:target
GvHD: Graft versus host disease
HSC: Hematopoietic stem cell
HSPC: Hematopoietic stem/progenitor cell
ICANS: Immune effector cell-associated neurotoxicity syndrome
LSC: Leukemic stem cell
NK: Natural killer
NSG: NOD SCID gamma
O/E: Overexpression
WT: Wild type
