# Supplementary material for: Anti-TIM3 chimeric antigen receptor-natural killer cells preferentially target primitive acute myeloid leukemia cells with minimal fratricide and exhaustion
Source: Exp Hematol Oncol. 2024 Jul 11;13:67. doi: 10.1186/s40164-024-00534-2 (PMC11238396; doi:10.1186/s40164-024-00534-2)
Supplement: Supplementary file 1 — Additional file 1: Methods. [file 40164_2024_534_MOESM1_ESM.pdf]

Anti-TIM3 chimeric antigen receptor-natural killer cells preferentially target primitive acute myeloid leukemia cells with minimal fratricide and exhaustion

Phatchanat Klaihmon, Parinya Samart, Yon Rojanasakul, Surapol Issaragrisil, Sudjit Luanpitpong

Correspondence: Sudjit Luanpitpong, Siriraj Center of Excellence for Stem Cell Research, Faculty of Medicine Siriraj Hospital, Mahidol University, 2 Siriraj Hospital, Bangkoknoi, Bangkok 10700, Thailand; Tel.: +66 2 419 2907; Email: suidjit@gmail.com.

Additional file 1

Methods

Subjects and ethics statement

This study was approved by the Siriraj Institutional Review Board (COA No. Si 755/2022) and was in accordance with the Helsinki Declaration of 1975. Human specimens were collected after written informed consents were obtained.

Data mining and database analyses

Analyses of mRNA expression of *HAVCR2* were performed using BloodPool: AML samples with normal cells (probe 1555628_a_at) in BloodSpot database (https://www.bloodspot.eu/) [1] and TCGA dataset in UALCAN database by the University of Alabama at Birmingham (https://ualcan.path.uab.edu/) [2]. Gene Set Enrichment Analysis (GSEA; v4.3.3) was used to analyze the differences in gene expression between leukemic stem cells (LSCs), leukemic progenitor cells (LPCs), and normal hematopoietic stem cells (HSCs) using GSE24006 dataset [3].

Cell lines and cell culture

All cell lines, including effector NK-92 cells and target tumor Kasumi-3, KG-1, Kasumi-1, U937, and HEL92.1.7 cells were purchased from American Type Cell Collection (ATCC; Manassas, VA, USA). Briefly, AML-derived cell lines were cultured in complete RPMI 1640 medium, while NK-92 cells were grown in MEM-α medium supplemented with 100 U/ml IL-2 as per ATCC's recommendations at 37 °C, 5% CO_2 and 95% relative humidity. Mycoplasma contamination was checked every 3 months by MycoAlert Plus mycoplasma detection kit (Lonza, Cologne, Germany), and any cell lines found positive were discarded.

Patient-derived primary AML cells

Cryopreserved patient-derived primary AML cells, which were obtained from peripheral blood mononuclear cells of a patient, were thawed in the presence of DNase. Cells were then cultured in StemSpan medium (Stem Cell Technologies, Vancouver, Canada) supplemented with 10 ng/mL IL-3 and G-CSF, 25 ng/ mL TPO, and 50 ng/mL SCF and FLT3 ligand (R&D Systems, Minneapolis, MN, USA).

Collection of hematopoietic stem/progenitor cells

Normal hematopoietic stem/progenitor cells (HSPCs) were freshly collected from G-CSF mobilized peripheral blood of healthy donors using CD34 MicroBead Kit and MS Column (Miltenyi Biotec, Bergisch Gladbach, Germany) according to the manufacturer protocol. The cells were cultured in StemSpan medium (Stem Cell Technologies, Vancouver, Canada) supplemented

with 50 ng/mL SCF, 50 ng/mL FLT3 ligand, 25 ng/mL TPO, 10 ng/mL IL-3 and 10 ng/mL G-CSF (R&D Systems, Minneapolis, MN, USA) for 3 days before use in NK cell cytotoxicity assay.

Collection of peripheral blood NK cells

Mononuclear cells were isolated from 10 mL fresh peripheral blood (PB) of healthy donors using Ficoll-Paque Premium solution (Cytiva, Marlborough, MA, USA) and stained with FITC-conjugated anti-CD3, PE-conjugated anti-CD56, and PerCP-Cy5.5-conjugated anti-CD16 antibodies (BD Biosciences, San Jose, CA, USA) prior fluorescence-activated cell sorting (FACS) with FACS Aria Fusion cell sorter (BD Biosciences). Sorted PB-NKs, were cultured in NK MACS cell medium (Miltenyi Biotech) plus 1% NK MACs supplement, 5% AB serum and 500 IU/ml IL-2 for 3 days

Lentiviral production and transduction of NK cells

Sequences of the third-generation CAR targeting TIM3 with CD3ζ signaling domain and costimulatory domains CD28 and 4-1BB (CAR-TIM3) were synthesized and cloned into a lentiviral vector with EF1α promoter by Creative Biolabs (Shirley, NY, USA). The anti-TIM3 scFv fragment (clone TSR-022) used was as follows: DIQMTQSPSSLSASVG DRVTITCRASQSIRRYLNWYHQKPGKAPKLLIYGASTLQSGVPSRFSGSGSGTDFTLTISS LQPEDFAVYYCQQSHSAPLTFGGGTKVEIKGGGGSGGGGSGGGGSEVQLLESGGGLVQ PGGSLRLSCAAASGFTFSSYDMSWVRQAPGKGLDWVSTISGGGTYTYYQDSVKGRFTIS RDNSKNTLYLQMNSLRAEDTAVYYCASMDYWGQGTTVTVSS. Lentiviral particles were produced in HEK293FT cells (ATCC) by cotransfection of CAR-TIM3 with p-VSVG and dR8.2 plasmids at the ratio of 10:1:4 using Lipofectamine 3000 reagent (Thermo Fisher Scientific,

Waltham, MA, USA). The viral supernatants were harvested after 48 h, filtered through a 0.45-μm filter membrane, and then centrifuged using Amicon Ultra-15 centrifugal filter tube (Merck Millipore, Tullagreen, Ireland) at 4,500 ×g for 1 h.

To introduce the CAR-TIM3 transgene, NK-92 cells and peripheral blood NK cells at the density of 5×10^5 NK cells per 0.5 mL were mixed with concentrated viral particles in the presence of 8 μg/mL polybrene and spinoculated at 2,250 rpm for 1 h at 32 °C, after which, 0.5 mL medium were added. At 24h posttransduction, the cells were replenished with fresh medium, grown, and FACS-sorted based on the positivity to anti-human F(ab')2 antibody (Jackson ImmunoResearch, West Grove, PA, USA) to enrich CAR-TIM3-positive NK cells using FACS Aria Fusion cell sorter (BD Biosciences, San Jose, CA, USA). Cell transduction and sorting were repeated thrice to ensure that more than 80% of the cells in culture were positive for F(ab')2.

To ascertain TIM3 binding activity, cells were incubated with 10 μg/mL rhTIM3 (23–200) protein with His tag to the C-terminus (Abcam, Cambridge, UK) for 1 h at 4 °C, followed by an incubation with FITC-conjugated anti-His tag antibody (His tag-FITC; Abcam) for 15 min at room temperature. Cells that were incubated with His tag-FITC, but not with rhTIM3, were used as a basal control.

RT-qPCR analysis of CD3ζ and scFv fragment

Total RNA was isolated using TRI Reagent (Molecular Research Center, Cincinnati, Ohio, USA) and converted to complementary DNA using the RevertAid First Strand cDNA synthesis kit (Thermo Fisher Scientific). The qPCR reactions were performed on the CFX384 Touch Real-Time PCR detection system (Bio-Rad, Hercules, CA, USA) using SYBR Select Master Mix (Thermo Fisher Scientific). The cycle parameters started with an activation step at 95 °C for 2 min,

followed by 40 cycles of denaturation at 95 °C for 15 s and annealing/extension at 60 °C for 1 min. Transcription abundance (arbitrary unit) was calculated by the $2^{-\Delta Ct}$. The following primers were used: *CD3ζ* forward: 5'-GAGTACGACGTGCTGGACAA-3' and reverse: 5'-GCGTCSTAGGTGTCCTTGGT-3'; *scFv* forward: 5'-CACATGTAGAGCCAGCCAGA-3' and reverse: 5'-GGGAGGAGATGGTCAGTGTG-3'; *GAPDH* forward: 5'-AGCCACATCGCTCAGACAC-3' and reverse: 5'-GCCCAATACGACCAAATCC-3'.

Western blotting

Cells were pelleted and lysed using RIPA buffer (Cell Signaling Technology, Danvers, MA, USA). The cell lysates were clarified by centrifugation, and protein was quantified using BCA assay (Pierce Biotechnology, Rockford, IL, USA). Equal amounts of protein were loaded and separated by SDS-PAGE under reducing conditions and transferred to a PVDF membrane. Next, the membrane was incubated with mouse anti-CD3ζ antibody (1:1000; BD Biosciences) followed by an HRP-labeled goat anti-mouse IgG secondary antibody (1:5000; Sigma Aldrich, St. Louis, MA, USA). The immune complex was detected by the Immobilon® Western Chemiluminescent HRP substrate (Millipore, Burlington, MA, USA).

Immunophenotypic analysis

For immunophenotypic analysis of AML cells, cells were blocked in 2% BSA in PBS prior to incubation with FITC-conjugated anti-CD34, PE-conjugated anti-CD38, PerCP-conjugated anti-CD45, and APC-conjugated anti-TIM3 antibodies. PE/Vio770-conjugated anti-PD-1 (Miltenyi Biotec, Bergisch Gladbach, Germany) was additionally used to evaluate NK cell exhaustion. The mixture was incubated for 30 min at 4 °C in the dark, and subjected to flow cytometric analysis

using BD FACS Canto cytometer (BD Biosciences). The acquired data were analyzed using FlowJo software, version 10.4.1 (FlowJo, Ashland, OR, USA).

NK cell cytotoxicity assay

For functional analysis, target AML cells were fluorescently pre-labeled with PKH67 dye (Sigma-Aldrich) according to the manufacturer's instructions. After which, wild type (WT) or CAR-TIM3 NK-92 cells were co-cultured with different numbers of PKH67-labeled AML cells at effector:target (E:T) ratios of 1:1, 1:2, 1:5, and 1:10 in round bottom 96-well plate for a total cells of 10^5 cells per microwell for 4 h at 37 °C . Target cell death was then evaluated by annexin-V/7-AAD assay. Briefly, cell pellets were stained with annexin-V and 7-AAD (BD Biosciences) in binding buffer containing optimum calcium chloride for 15 min in the dark before being subjected to flow cytometric analysis of PKH67-positive cells using BD FACS Canto flow cytometer.

Cytokine release analysis

CAR-TIM3 and WT NK-92 cells were co-cultured with target tumor cells at the E:T ratio of 1:1 for 24 h, after which the levels of human IFN-γ and TNF-α in the supernatant were measured using ELISA MAX Deluxe Set Human Kit (BioLegend) according to the manufacturer's instructions.

Colony formation assay

AML cells were co-cultured with CAR-TIM3 or WT NK-92 cells at E:T ratios of 1:1, 1:5 and 1:10 for 4 h. Then, 300 cells from each group were seeded in 0.4 mL MethoCult H4100 in a 24-well

plate. After 10 days, the emerging hematopoietic colonies were visualized and scored under an inverted microscope (Eclipse Ti-U, Nikon, Tokyo, Japan).

Xenotransplantation mouse model

All animal experiments were approved by the Institutional Animal Care and Use Committee (IACUC #1602000428_R1.1) and were performed in accordance with the Guidelines for Animal Experiments at West Virginia University. NOD.Cg-*Prkdc*^{scid} *Il2rg*^{tm1Wjl} /SzJ (NOD SCID gamma, NSG) mice were obtained from WVU Transgenic Animal Core Facility and housed under conventional barrier protection. Luciferase (Luc)-labeled TIM3-overexpressed (O/E) U937 AML cells at 1.5×10^6 cells were intravenously (I.V.) injected into female NSG mice aged 6−8 weeks. Two doses of 1.5×10^6 CAR-TIM3 or WT NK-92 cells were administered via I.V. injection on the days 7 and 11 after AML inoculation. To evaluate tumor growth of Luc2-labeled cells, each mouse received 150 mg/kg of XenoLight D-luciferin K^+ salt (Perkin Elmer, Waltham, MA, USA) via intraperitoneal (I.P.) injection and monitored every 3−4 days using IVIS Spectrum in vivo imaging system (Perkin Elmer). The biolouminescent images were processed and analyzed using Living Image 3.2 software (Perkin Elmer). Bone marrow was dissected from the femur bone of hind limbs, and flow cytometric analysis of human $CD45^+$ and $TIM3^+$ cells was performed to identify the residual human AML cells.

Statistical analysis

All experiments were performed in triplicate. Data are expressed as mean ± SD from independent experiments. Statistical analysis of the data was determined by Student's *t*-test, Mann–Whitney U-

test, or one-way ANOVA followed by a Bonferroni post-test. A p-value < 0.05 was considered statistically significant.

References

1. Bagger FO, Sasivarevic D, Sohi SH, Laursen LG, Pundhir S, Sønderby CK, et al. BloodSpot: a database of gene expression profiles and transcriptional programs for healthy and malignant haematopoiesis. Nucleic Acids Res. 2016;44(D1):D917−24.
2. Chandrashekar DS, Karthikeyan SK, Korla PK, Patel H, Shovon AR, Athar M, et al. UALCAN: An update to the integrated cancer data analysis platform. Neoplasia. 2022;25:18−27.
3. Gentles AJ, Plevritis SK, Majeti R, Alizadeh AA. Association of a leukemic stem cell gene expression signature with clinical outcomes in acute myeloid leukemia. JAMA. 2010;304(24):2706−15.
