# Supplementary material for: Anti-TIM3 chimeric antigen receptor-natural killer cells preferentially target primitive acute myeloid leukemia cells with minimal fratricide and exhaustion
Source: Exp Hematol Oncol. 2024 Jul 11;13:67. doi: 10.1186/s40164-024-00534-2 (PMC11238396; doi:10.1186/s40164-024-00534-2)
Supplement: Supplementary file 2 — Additional file 2: Fig. S1 Analysis of HAVCR2 gene expression in clinical AML specimens using available public databases. Fig. S2 Ranked gene list correlation profile for LSCs versus LPCs or normal HSCs by Gene Set Enrichment Analysis (GSEA) using GSE24006 dataset. Fig. S3 Flow cytometric analysis of surface TIM3 expression in different subpopulations of leukocytes obtained from AML and normal whole blood samples. Fig. S4 Validation of CAR expression in NK-92 cells based on target antigen-based binding and activation. Fig. S5 Anti-AML activity of CAR-TIM3 NK92 cells against primary AML cells. Fig. S6 Overexpression of TIM3 in AML cell lines with relatively more mature phenotype. Fig. S7 Pro-inflammatory cytokines released by CAR-TIM3 NK-92 cells upon AML exposure. Fig. S8 Flow cytometric analysis of surface NK cell activating receptors, including Nkp44, Nkp46, and NKG2D, in WT and CAR-TIM3 NK-92 cells. Fig. S9 Anti-AML activity of peripheral blood-derived CAR-TIM3 NK cells against various AML cells. Fig. S10 TIM3 mediates NK cytotoxicity against primary AML cells. [file 40164_2024_534_MOESM2_ESM.pdf]

Anti-TIM3 chimeric antigen receptor-natural killer cells preferentially target primitive acute myeloid leukemia cells with minimal fratricide and exhaustion

Phatchanat Klaihmon, Parinya Samart, Yon Rojanasakul, Surapol Issaragrisil, Sudjit Luanpitpong

Correspondence: Sudjit Luanpitpong, Siriraj Center of Excellence for Stem Cell Research, Faculty of Medicine Siriraj Hospital, Mahidol University, 2 Siriraj Hospital, Bangkoknoi, Bangkok 10700, Thailand; Tel.: +66 2 419 2907; Email: suidjit@gmail.com.

Additional file 2

Fig. S1−S10.

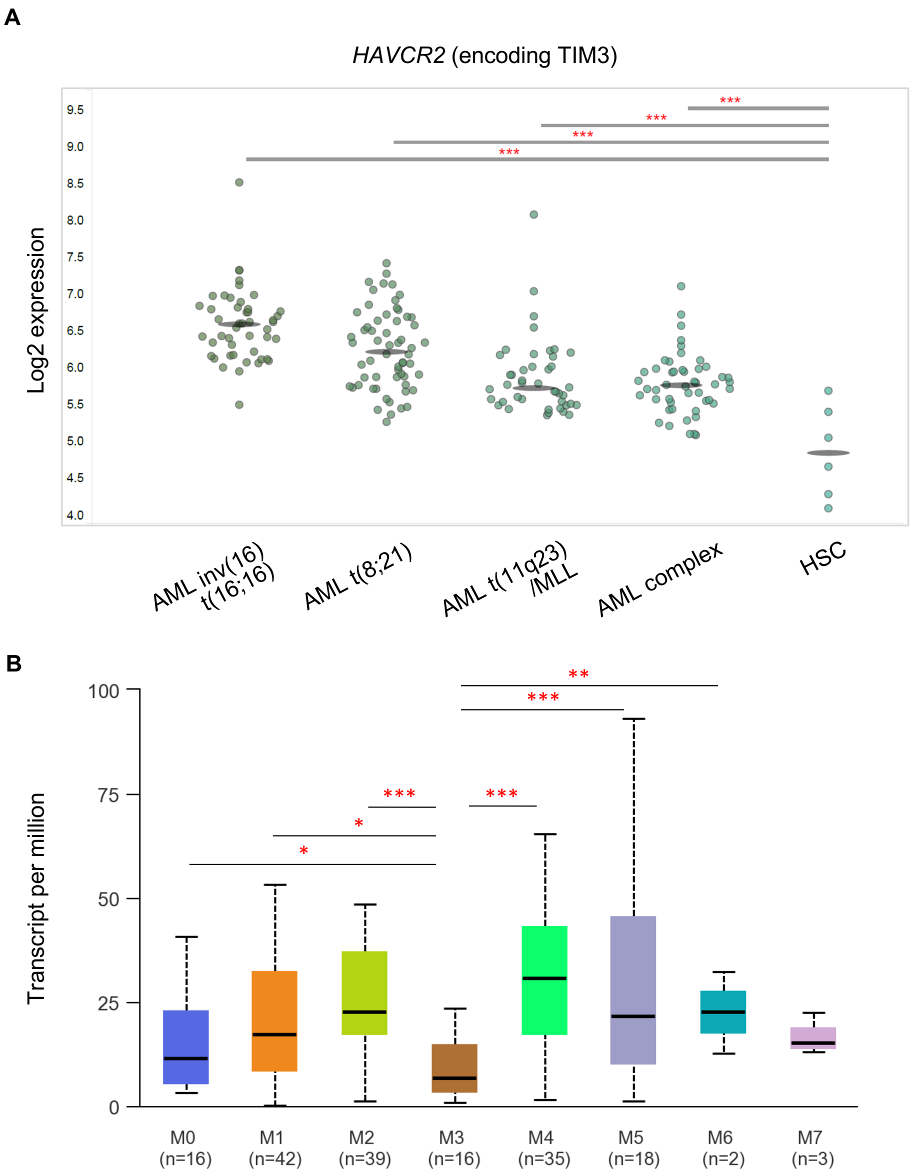

Fig. S1 Analysis of *HAVCR2* gene expression in clinical AML specimens using available public databases. (A) *HAVCR2* expression in AML samples with different molecular cytogenetic abnormalities compared to normal HSCs using BloodPool dataset in BloodSpot database. $^{***}p < 0.001$; Student's *t*-test. (B) *HAVCR2* transcript level was analyzed in AML specimens based on FAB classification using UALCAN database. $^{*}p < 0.05$; $^{**}p < 0.01$; $^{***}p < 0.001$; Student's *t*-test.

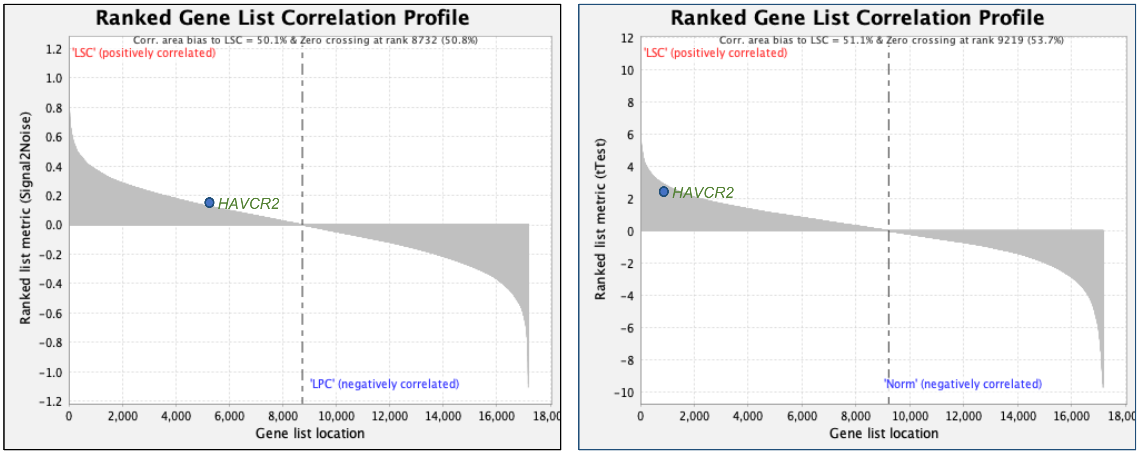

Fig. S2 Ranked gene list correlation profile for LSCs versus leukemic progenitor cells (LPCs) or normal HSCs by Gene Set Enrichment Analysis (GSEA) using GSE24006 dataset. *HAVCR2* expression ranks as LSCs > LPCs >> HSCs.

A

	PB-AML #2
Sex/Age	Female/43 years
RBC count	2.82 x 10^6/uL
Hb	7.6 g/dL
Hct	22.8 %
Plt count	14 x 10^3/uL
WBC count	1.61 x 10^6/uL
Immuno-phenotyping	$CD13^+$, $CD33^+$, $CD34^+$, $CD117^+$, $CD7^+$, HLA-DR^+

B

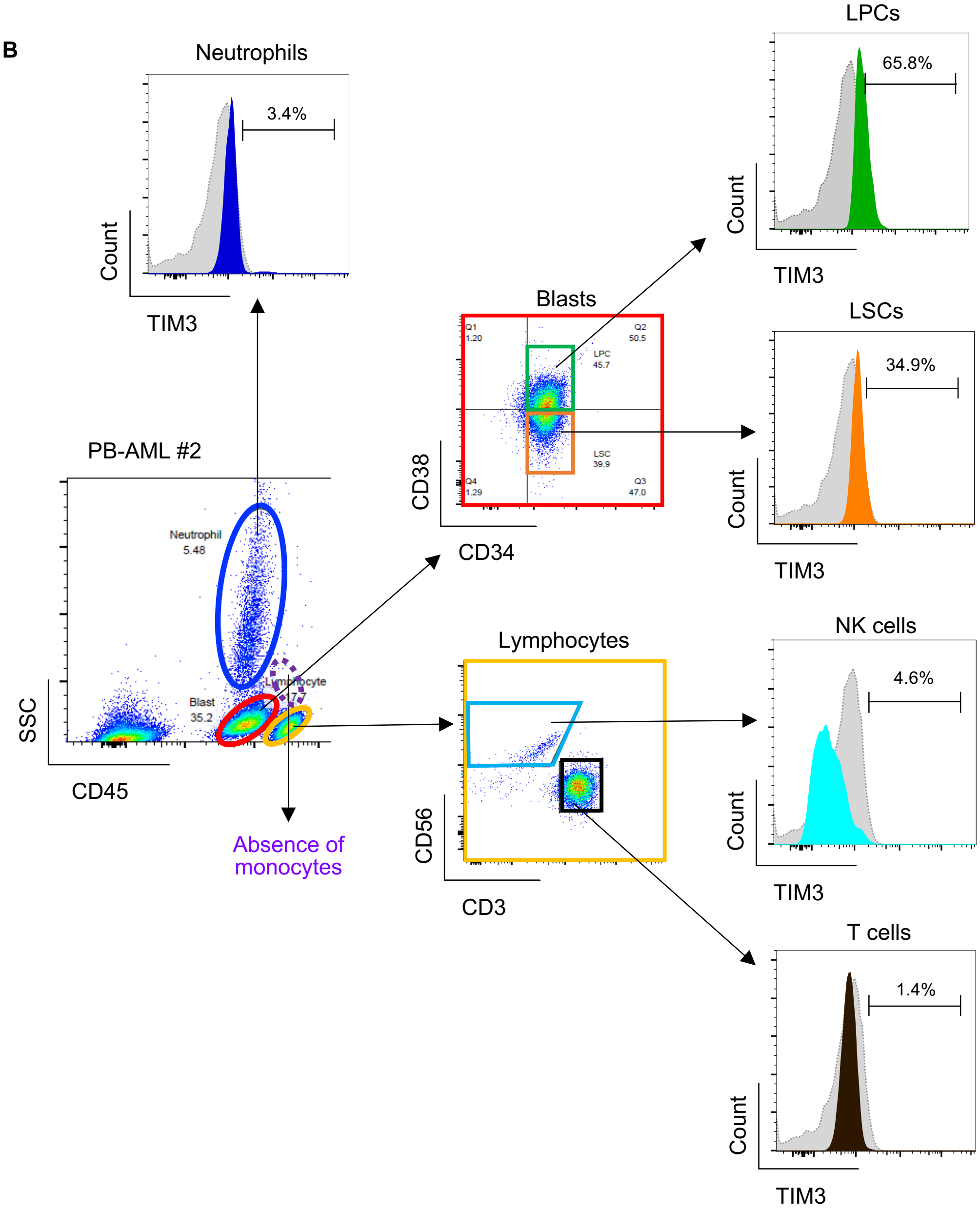

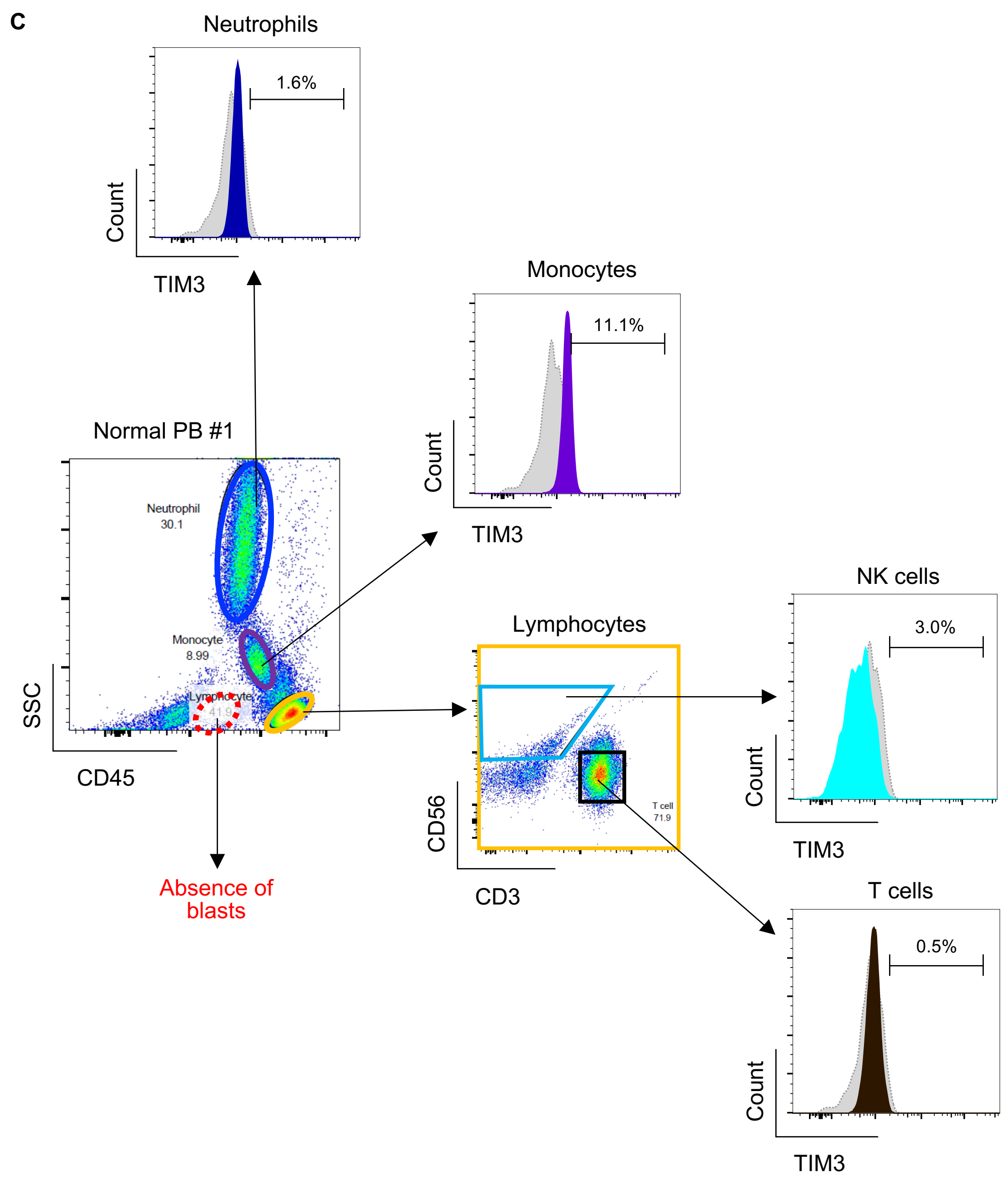

Fig. S3 Flow cytometric analysis of surface TIM3 expression in different subpopulations of leukocytes obtained from AML and normal whole blood samples. (A) Clinical characteristics of AML patient-derived whole blood. (B, C) Flow cytometric analysis of surface TIM3 expression in different leukocytes subpopulations obtained from AML (B) and normal (C) whole blood samples, including neutrophils, monocytes (absent in AML), $CD3^{-}CD5^{+}$ NK cells, and

$CD3^{+}CD56^{-}$ T cells. AML cells were $CD34^{+}CD38^{-}$ LSCs and $CD34^{+}CD38^{+}$ LPCs, both were $TIM3^{+}$. The stained sample was overlaid onto the IgG1 isotype control (grey histogram).

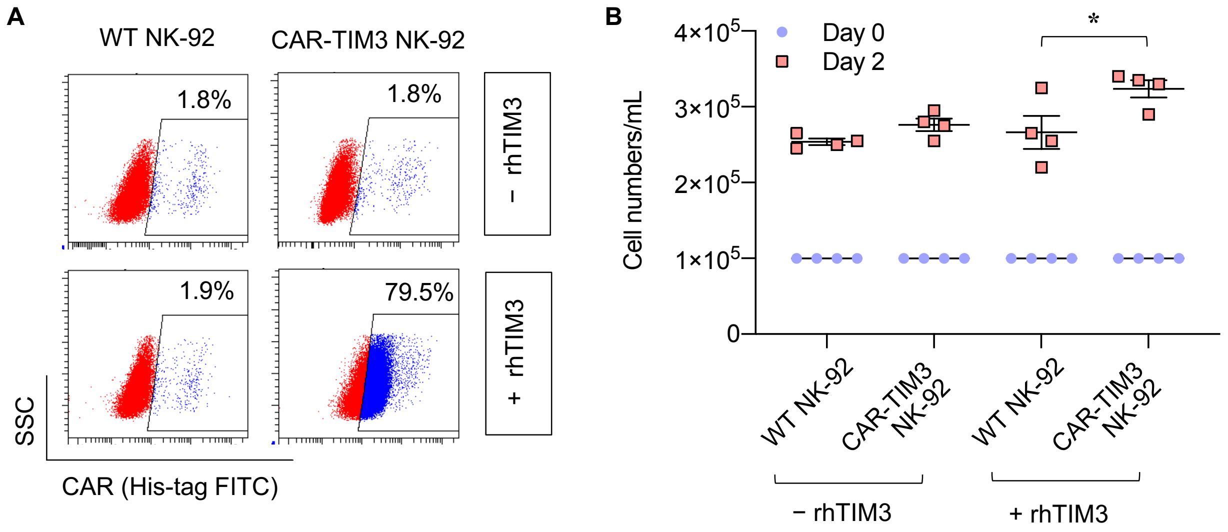

Fig. S4 Validation of CAR expression in NK-92 cells based on target antigen-based binding and activation. (A) CAR-TIM3 expression (box) in WT and CAR-TIM3 NK-92 cells as evaluated by flow cytometry based on its binding activity to the specific antigen His tag-rhTIM3. (B) Cell proliferation in WT and CAR-TIM3 NK-92 cells after activation with rhTIM3 for 2 days. $^{*}p < 0.05$; Mann–Whitney U-test.

A

	PB-AML #1
Sex/Age	Female/82 years
RBC count	3.11 x 10^6/uL
Hb	8.1 g/dL
Hct	26.1 %
Plt count	66 x 10^3/uL
WBC count	62 x 10^3/uL
Immuno-phenotyping	$CD13^+$, $CD33^+$, $CD11c^+$, $CD117^+$, $HLA\text{-}DR^+$, $CD34^+$, $CD56^+$, $CD71^+$

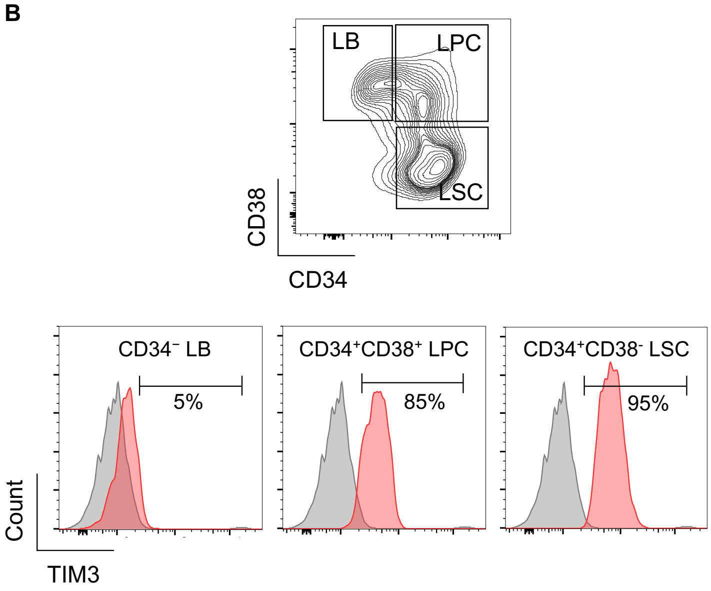

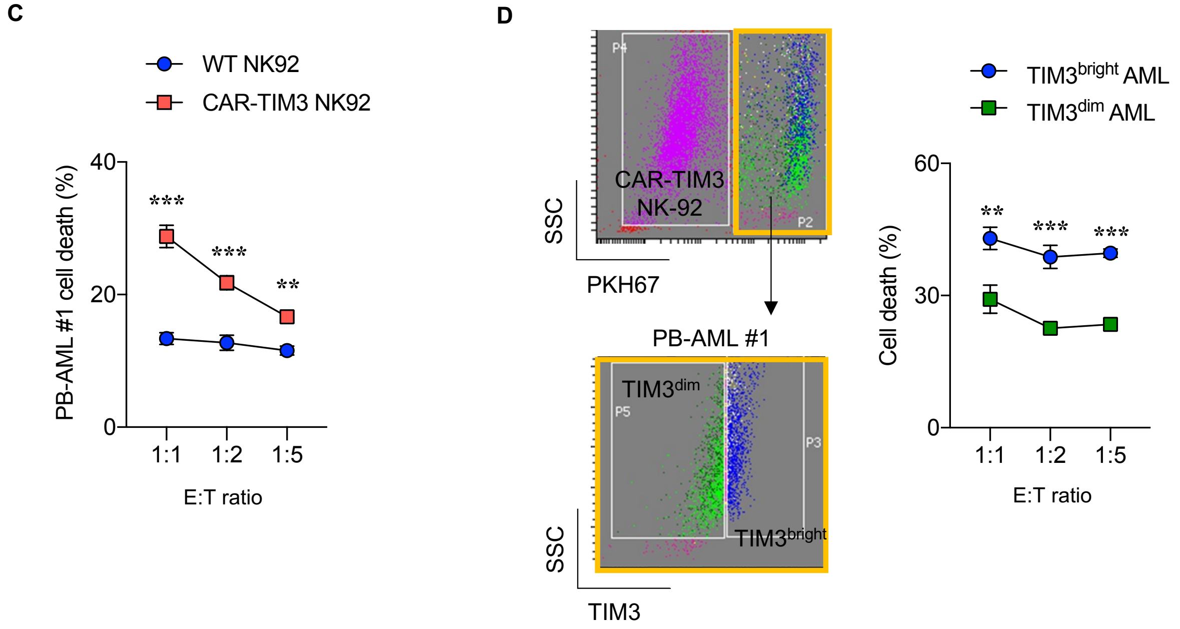

Fig. S5 Anti-AML activity of CAR-TIM3 NK92 cells against primary AML cells. (A) Clinical characteristics of AML patient-derived primary cells. (B) Flow cytometric analysis of surface TIM3 expression in different AML subpopulations, $CD34^+CD38^-$ LSCs, $CD34^+CD38^+$ LPCs, and $CD34^-$ leukemic blasts (LBs). (C) Percentages of total cell death of PKH67-labeled PB-AML #1 cells after exposure to either unlabeled WT or CAR-TIM3 NK-92 cells for 6 h by annexin-V/7-

AAD assay. Basal death rate (without NK cells) was subtracted from all data shown. $^{**}p < 0.01$, $^{***}p < 0.001$ vs WT NK cells at the same E:T ratio; Mann–Whitney U-test. (D) Percentages of total cell death of PKH67-labeled PB-AML #1 cells in response to CAR-TIM3 NK-92 cells at 6 h according to the level of TIM3 expression (dim or bright). $^{**}p < 0.01$, $^{***}p < 0.001$ vs TIM3^{dim} cells at the same E:T ratio; Mann–Whitney U-test.

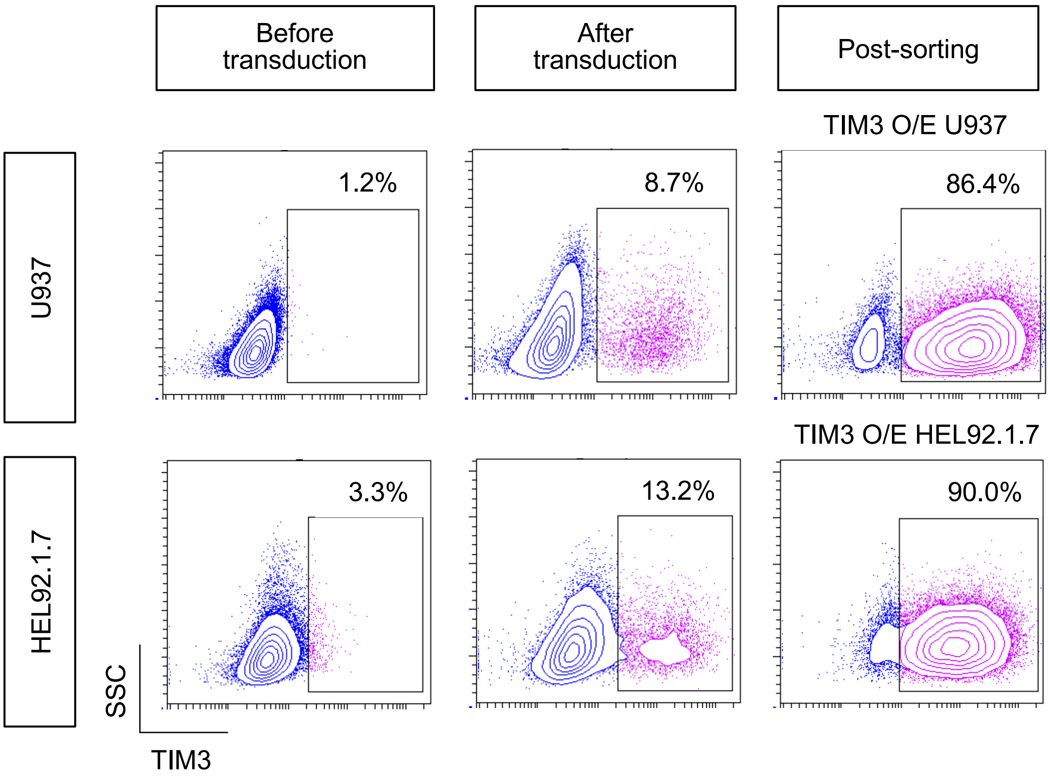

Fig. S6 Overexpression of TIM3 in AML cell lines with relatively more mature phenotype. AML U937 (FAB M5) and HEL92.1.7 (M6) cells were transduced with retroviral particles carrying myc-tagged human TIM3 (Addgene #110893) and enriched for TIM3-overexpressed (O/E) cells using FACS. Expression of surface TIM3 in TIM3 O/E U937 and HEL92.1.7 cells was shown.

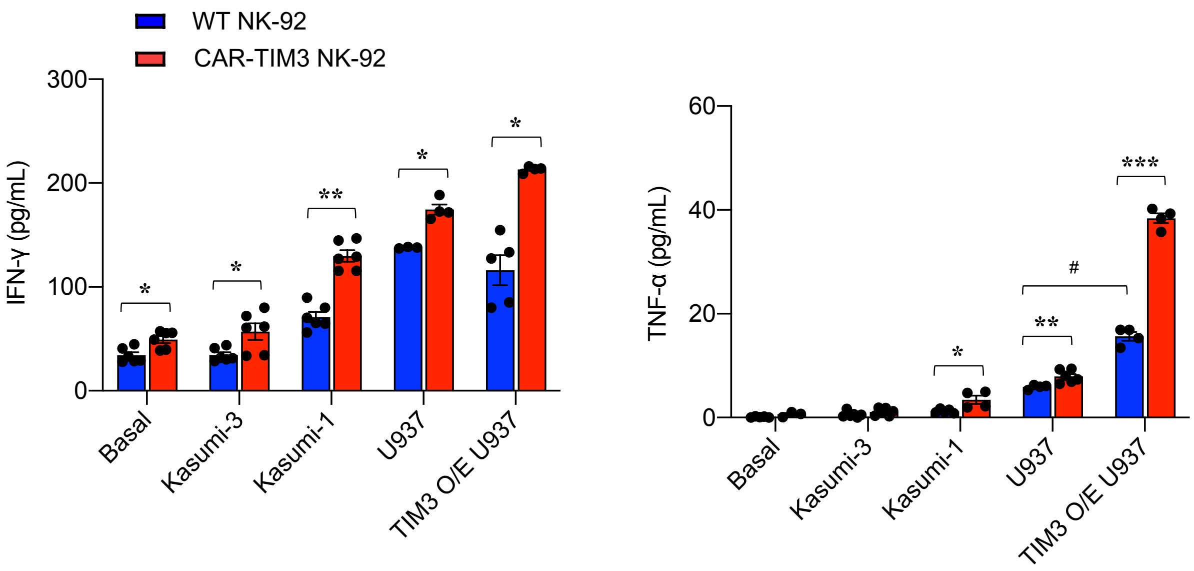

Fig. S7 Pro-inflammatory cytokines released by CAR-TIM3 NK-92 cells upon AML exposure. Quantitative measurement of IFN-γ (left) and TNF-α (right) by ELISA in cell-free supernatant collected from the coexposure of WT or CAR-TIM3 NK-92 cells to AML cells at E:T ratio of 1:1 at 24 h. $^{*}p < 0.05$, $^{**}p < 0.01$, $^{***}p < 0.001$ vs WT NK-92 cells; $^{\#}p < 0.05$ vs WT NK-92 cells coexposure to mock U937 cells; Mann–Whitney U-test.

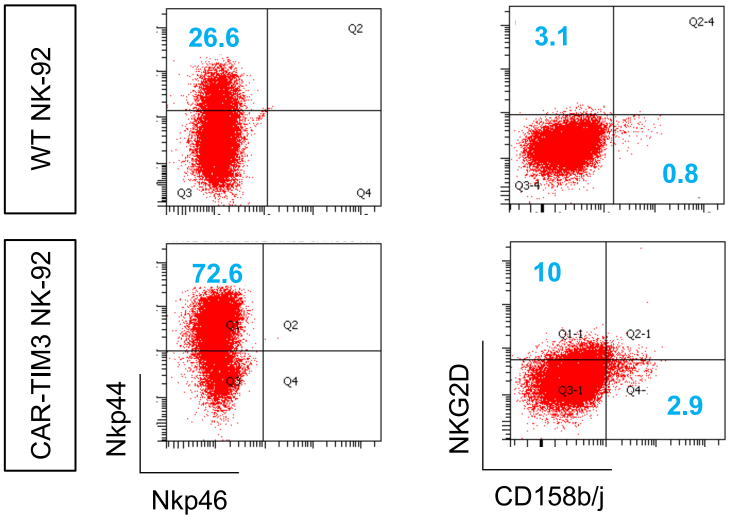

Fig. S8 Flow cytometric analysis of surface NK cell activating receptors, including Nkp44, Nkp46, and NKG2D, in WT and CAR-TIM3 NK-92 cells.

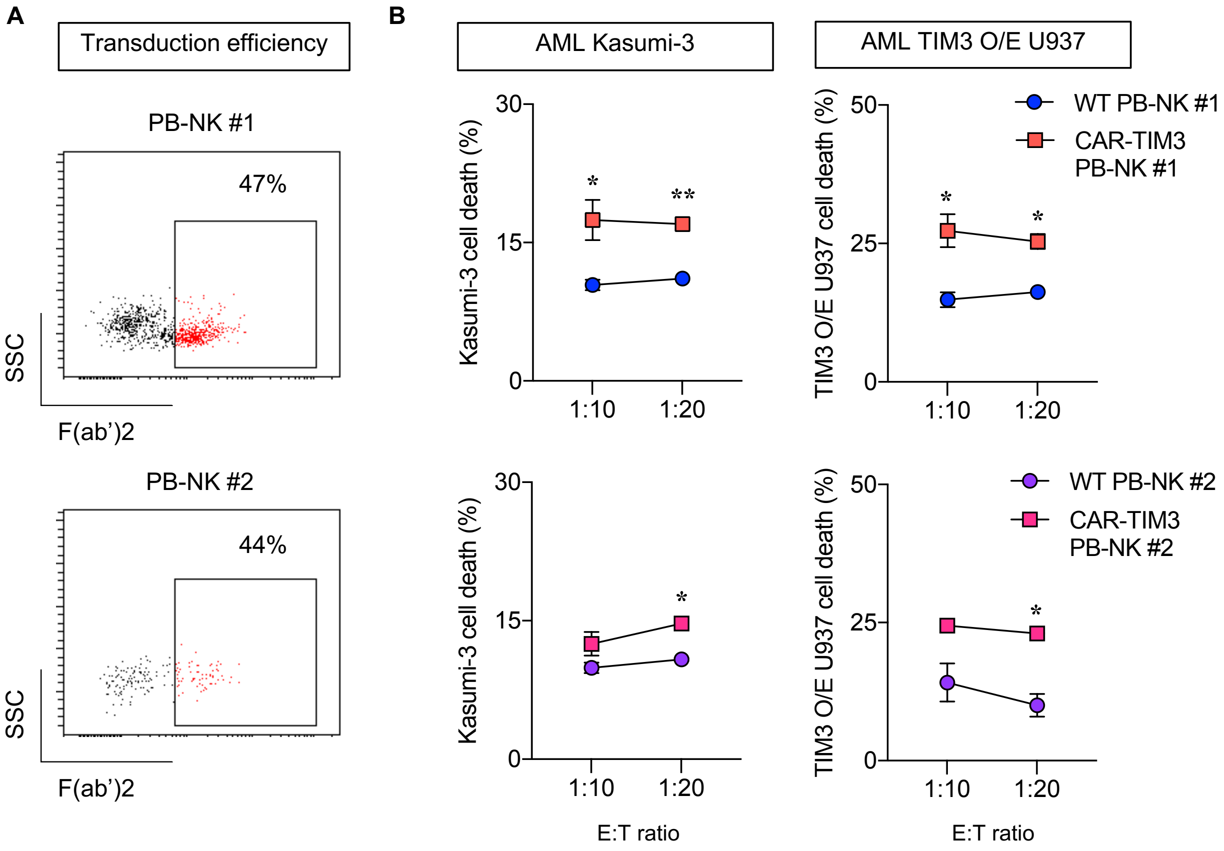

Fig. S9 Anti-AML activity of peripheral blood-derived CAR-TIM3 NK cells against various AML cells. (A) Transduction efficiency of CAR-TIM3 in peripheral blood (PB)-NK cells obtained from healthy donors #1 and #2 as determined by the positivity to anti-F(ab')2 antibody. (B, C) Percentages of total cell death of PKH67-labeled Kasumi-3 (B) and TIM3-overexpressed (O/E) U937 (C) cells after exposure to either unlabeled WT or CAR-TIM3 PB-NK cells (#1 and #2) at different E:T ratios for 4 h by annexin V/7-AAD assay. $^{*}p < 0.05$, $^{**}p < 0.01$ vs WT PB-NK cells (#1 and #2) at the same E:T ratio; Mann–Whitney U-test.

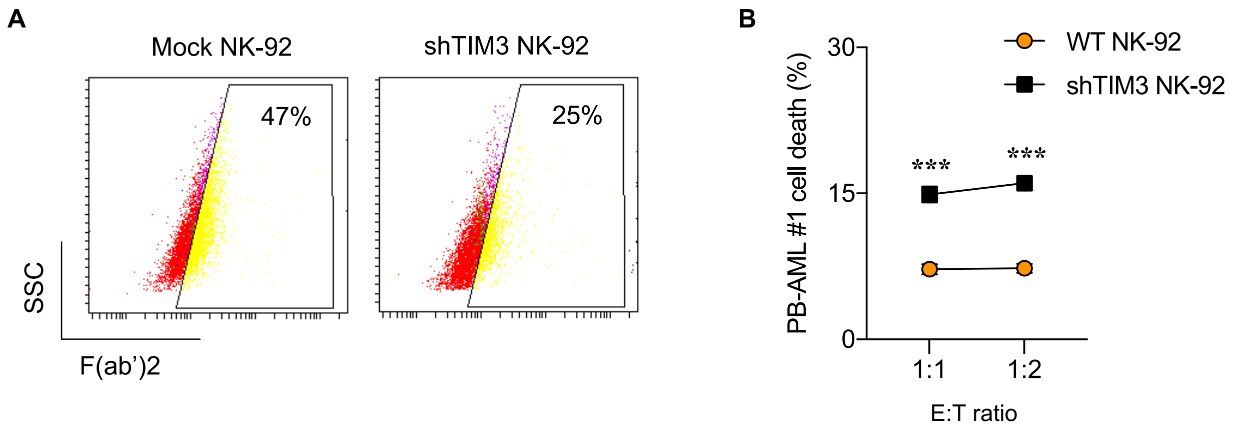

Fig. S10 TIM3 mediates NK cytotoxicity against primary AML cells. (A) TIM3 knockdown experiments were performed using NK-92 cells treated with lentiviral particles carrying pooled shRNAs against human *HAVCR2* (shTIM3) (#SC-72034-SH, Santa Cruz Biotechnology, Santa Cruz, CA, USA). Flow cytometric analysis of surface TIM3 in shTIM3 and mock NK-92 cells was shown. (B) Percentages of total cell death of PKH67-labeled PB-AML #1 cells after exposure to either unlabeled mock or shTIM3 NK-92 cells for 4 h by annexin-V/7-AAD assay. Basal death rate (without NK cells) was subtracted from all data shown. $^{***}p < 0.001$ vs mock NK-92 cells at the same E:T ratio; Mann–Whitney U-test.
